# Shaping the future of anesthesia research: celebrating progress and embracing new challenges

**DOI:** 10.1016/j.bjane.2024.844582

**Published:** 2024-12-12

**Authors:** Andre P. Schmidt

**Affiliations:** a Hospital de Clínicas de Porto Alegre (HCPA), Serviço de Anestesia e Medicina Perioperatória, Porto Alegre, RS, Brazil; b Universidade Federal do Rio Grande do Sul (UFRGS), Instituto de Ciências Básicas da Saúde (ICBS), Departamento de Bioquímica, Porto Alegre, RS, Brazil; c Universidade Federal de Ciências da Saúde de Porto Alegre (UFCSPA), Santa Casa de Porto Alegre, Serviço de Anestesia, Porto Alegre, RS, Brazil; d Hospital Nossa Senhora da Conceição, Serviço de Anestesia, Porto Alegre, RS, Brazil; e Universidade Federal do Rio Grande do Sul (UFRGS), Faculdade de Medicina, Programa de Pós-graduação em Ciências Pneumológicas, Porto Alegre, RS, Brazil; f Universidade Federal do Rio Grande do Sul (UFRGS), Programa de Pós-graduação em Ciências Cirúrgicas, Porto Alegre, RS, Brazil; g Faculdade de Medicina da Universidade de São Paulo (FMUSP), Programa de Pós-Graduação em Anestesiologia, Ciências Cirúrgicas e Medicina Perioperatória, São Paulo, SP, Brazil

The Brazilian Journal of Anesthesiology (BJAN), originally founded as the “Revista Brasileira de Anestesiologia” in 1951, has been a cornerstone of scientific advancement in anesthesiology for over seven decades. Initially launched in Portuguese, it transitioned to full English publication to align with global scientific communication standards and enhance its international reach. As one of the oldest continuously published anesthesiology journals, BJAN has embraced open access since its inception, supported by the Brazilian Society of Anesthesiology (SBA). Over time, the journal has evolved through innovations such as adopting modern editorial practices, fostering international collaborations, enhancing peer-review processes, and leveraging digital platforms to broaden its audience. Today, BJAN stands as a relevant platform for the dissemination of high-quality research in Latin America and globally, reflecting its commitment to excellence in medical science and education.

A journal's success relies on the content and quality of its published manuscripts. However, this success is never the work of one individual; rather, it is the collective achievement of a dedicated team. This team includes authors, peer reviewers, editors, board members, and readers. Each one has played a pivotal role in shaping the BJAN's identity and ensuring its relevance and impact in the field. The BJAN team sincerely thanks all its contributors for their indispensable roles in this process.

In particular, the reviewers’ contributions are fundamental to maintaining the journal's high standards. The reviewers, often working behind the scenes in a blinded peer-review process, perform the critical task of evaluating manuscripts, providing constructive remarks, and assisting editors in making informed decisions. Their scrutiny not only sharpens the quality of submissions but also ensures that published research is both rigorous and accessible to readers. The BJAN editorial office extends its gratitude to these invaluable collaborators, whose expertise and dedication form the backbone of the journal's scientific integrity.

BJAN has solidified its role as a premier journal in Latin America, serving as a critical platform for researchers from the region and the developing world to publish high-quality studies. Its Open Diamond model ensures free access for both authors and readers, providing an invaluable resource for disseminating scientific knowledge without financial barriers. The journal's commitment to accessibility has made it a basis for scientific advancement in anesthesiology, pain management, intensive care, and perioperative medicine across resource-limited settings. This openness fosters collaboration and ensures that groundbreaking research reaches practitioners and researchers who might otherwise lack access.

Recognizing the evolving needs of its audience, BJAN has embraced a paperless format, reflecting the habits of its readers who increasingly access content in digital formats. This shift has also been facilitated by the strategic partnership with Elsevier and the Editorial Manager platform, which have streamlined the processes of manuscript submission, review, and publication. These platforms enable seamless communication among authors, reviewers, and editors, ensuring efficient and transparent workflows.

Scientific integrity remains a key priority for BJAN and its editorial board. As fraud in science continues to threaten trust in research, the journal is committed to adopting stringent measures to detect and prevent unethical practices. Moving forward, BJAN has implemented enhanced plagiarism detection tools, promoted transparency through pre-registration of clinical trials, and encouraged authors to make raw data available in repositories. Additionally, BJAN has implemented strategies to educate reviewers and authors on best practices in ethical research and reporting, ensuring that publications meet the highest standards of integrity.

The journal recognizes the transformative role of artificial intelligence (AI) in modernizing editorial workflows. AI tools will increasingly assist in manuscript screening, plagiarism detection, and identifying potential conflicts of interest. These advancements will streamline peer-review processes and ensure efficient, high-quality evaluations. Furthermore, AI-driven analytics will provide insights into submission trends and help the journal adapt to emerging topics in anesthesiology and perioperative medicine.

BJAN has also developed robust social media strategies to enhance its visibility and impact. The creation of a Scientific Hub has enabled the production of educational content and facilitated discussions on scientific evidence and methodology. By joining platforms such as Instagram, YouTube, and Spotify, BJAN has expanded its reach, connecting with a global audience of researchers, clinicians, and students. These efforts have not only increased the visibility of published articles but also fostered a sense of community among professionals in anesthesiology.

Last year, BJAN adopted a continuous publication format, allowing for the timely dissemination of articles as soon as they are ready while still maintaining the tradition of organizing six curated issues per year. These issues are thoughtfully compiled to highlight the most relevant and impactful papers published in each cycle, with a focus on hot topics in anesthesia and related areas, including the publication of large observational studies,^1^, ^2^, ^3^, ^4^, ^5^ randomized clinical trials,^6^, ^7^, ^8^, ^9^ systematic reviews,^10^, ^11^, ^12^ and practice guidelines adapted to the reality of developing countries.^13^, ^14^, ^15^ Each issue also features editorials crafted by members of the editorial board or invited experts, offering in-depth commentary and insights on key advances and challenges within the field.^16^, ^17^, ^18^, ^19^, ^20^ These contributions reflect BJAN's commitment to addressing contemporary issues with scholarly rigor and clinical relevance.

In the upcoming editorial cycle, BJAN intends to implement more comprehensive statistical analyses for all accepted articles. The idea is for a dedicated team of statistical experts to review manuscripts, ensuring methodological robustness and data accuracy. This initiative aims to enhance the credibility and impact of published research. Additionally, to maintain and enhance the quality of its publications, BJAN plans to recruit and train a new generation of peer reviewers. Mentorship programs will be established to help reviewers develop critical evaluation skills, ensuring thorough and constructive feedback. By increasing the number and diversity of reviewers, BJAN will strengthen its peer-review process, reduce evaluation times, and support the publication of impactful research.

As my run comes to an end, it is time to pass the responsibility of editor-in-chief to someone whose skills and creativity are capable of meeting the journal's demands. It is an honor to introduce the next editor-in-chief, Professor Liana Maria Torres de Araújo Azi, an esteemed academic and anesthesiologist from the Federal University of Bahia (UFBA). Professor Liana Azi, the fourth woman in the last six editors of BJAN, symbolizes the growing prominence of female leadership in science. This transition reflects BJAN's and SBA's commitment to inclusivity and the promotion of women as scientific leaders. Her leadership promises to bring renewed vision and energy to the journal, continuing its mission of advancing anesthesiology and perioperative medicine.

The challenges that lie ahead for BJAN are both formidable and invigorating. The journal's future rests on sustaining high-quality publications, fostering ethical practices, embracing technological advancements, and engaging with readers across platforms. All of us look forward to working with the new editor-in-chief to advance the care of our patients and improve the scientific understanding in anesthesiology. I thank the authors, reviewers, readers, and the SBA for allowing me to have a wonderful professional experience as your editor-in-chief these last few years. With an unwavering commitment to excellence, BJAN will undoubtedly remain a reference of scientific integrity and innovation for Latin America and beyond.

## Conflicts of interest

The authors declare no conflicts of interest.
